# Expression of Stem Cell Markers in High-LET Space Radiation-Induced Intestinal Tumors in *Apc*^1638N/+^ Mouse Intestine

**DOI:** 10.3390/cancers15174240

**Published:** 2023-08-24

**Authors:** Elaina Kwiatkowski, Shubhankar Suman, Bhaskar V. S. Kallakury, Kamal Datta, Albert J. Fornace, Santosh Kumar

**Affiliations:** 1 Department of Biology, Georgetown University, Washington, DC 20057, USA; 2 Department of Oncology, Lombardi Comprehensive Cancer Center, Georgetown University Medical Center, Washington, DC 20057, USA; 3 Department of Pathology, Georgetown University Medical Center, Washington, DC 20057, USA; 4 Department of Biochemistry and Molecular & Cellular Biology, Georgetown University Medical Center, Washington, DC 20057, USA

**Keywords:** intestinal tumor, stem cells, space radiation, β-catenin, heavy-ion radiation, *Apc* ^1638N/+^

## Abstract

**Simple Summary:**

Exposure to ionizing radiation (IR) poses risks to the expanding interest in human space exploration; in atomic bomb survivors and radiological professionals, IR exposure has been linked to GI diseases, including colon cancer. Here, we assessed the expression of multiple stem cell markers in premalignant tumors and adjacent normal mucosa after low- and high-linear energy transfer (LET) irradiation. Our results revealed a noticeable correlation between increased levels of stemness markers and β-catenin activation in premalignant tumors, which was more prominent in tumors induced after exposure to high-LET radiation compared to low-LET induced tumors. Our findings also highlight the complex relationship between radiation types and stem cell phenotypes and their potential influence on carcinogenesis processes, which has significant implications for cancer risk assessments.

**Abstract:**

Estimation of cancer risk among astronauts planning to undertake future deep-space missions requires understanding the quantitative and qualitative differences in radiogenic cancers after low- and high-LET radiation exposures. Previously, we reported a multifold higher RBE for high-LET radiation-induced gastrointestinal (GI) tumorigenesis in *Apc*^1638N/+^ mice. Using the same model system, i.e., *Apc*^1638N/+^ mice, here, we report qualitative differences in the cellular phenotype of low- and high-LET radiation-induced GI tumors. Stem cell (SC) phenotypes were identified using BMI1, ALDH1, CD133, DCLK1, MSI1, and LGR5 markers in low (γ-rays)- and high (^56^Fe)-LET radiation-induced and spontaneous tumors. We also assessed the expression of these markers in the adjacent normal mucosa. All six of these putative SC markers were shown to be overexpressed in tumors compared to the adjacent normal intestinal tissue. A differential SC phenotype for spontaneous and radiogenic intestinal tumors in *Apc*^1638N/+^ mice was observed, where the ALDH1, BMI1, CD133, MSI1, and DCLK1 expressing cells were increased, while LGR5 expressing cells were decreased in ^56^Fe-induced tumors compared to γ-ray-induced and spontaneous tumors. Furthermore, higher β-catenin activation (marked by nuclear localization) was observed in ^56^Fe-induced tumors compared to γ and spontaneous tumors. Since differential tumor cell phenotype along with activated β-catenin may very well affect malignant progression, our findings are relevant to understanding the higher carcinogenic risk of high-LET radiation. This study has implications for the assessment of GI-cancer risk among astronauts, as well as for the estimation of secondary cancer risk among patients receiving hadron therapy, considering that our results indicate increased stemness properties after radiation.

## 1. Introduction

Astronauts are likely to receive a significant cumulative radiation dose from galactic cosmic radiation (GCR) during long-duration deep space missions. Particularly, the high atomic number and energy (HZE) particles present in GCR are considered a major threat to astronauts’ health as they can penetrate through spacecraft shielding and are densely ionizing due to their high linear energy transfer (LET) characteristics [1]. Exposure to HZE radiation results in higher levels of DNA damage, increased cell death as well as higher frequency of tumors, relative to the same dose of low-LET radiation, indicating a higher relative biological effectiveness (RBE) for these parameters [2,3]. Earlier, using adenomatous polyposis coli gene (*Apc*)-mutant mouse models of gastrointestinal (GI) tumorigenesis (*Apc^Min/+^* and *Apc*^1638N/+^ mice), we have reported a higher RBE for intestinal tumorigenesis, and higher incidence of carcinoma after HZE and simulated GCR exposure, relative to low-LET radiation [4,5,6,7]. Furthermore, altered cellular homeostasis, decreased cellular differentiation, increased cell transformation, epithelial cell proliferation, altered cell polarity/migration, and activation of oncogenic β-catenin signaling were observed and have also been implicated for higher tumorigenesis in HZE-exposed mouse intestine [5,8,9]. However, the mechanism(s) involved in the higher frequency of adenoma (benign tumor) to carcinoma (invasive cancer) progression after HZE exposure is not fully understood.

Intestinal stem cells (ISC) are considered the cell of origin for tumor initiation [10,11]. IR-induced malignant transformation of ISC is attributed to their capacity to self-renew, de-differentiate, and undergo mutagenesis [12,13]. Adenoma-to-carcinoma progression in gastrointestinal cancers follows a gradual increase in the heterogeneity of the SC population, while tumors with relatively higher stem-cell heterogeneity frequently progress to carcinoma [14]. Therefore, elucidating SC phenotypes in low-LET vs. HZE-induced tumors is important to understand the cellular mechanisms involved in the higher frequency of adenoma to carcinoma progression after HZE exposure. Several intestinal epithelial stem cells are identified by the characteristic expression of marker proteins, such as aldehyde dehydrogenase 1 (ALDH1), B lymphoma Mo-MLV Insertion Region 1 Homolog (BMI1), Prominin 1 (CD133), Doublecortin Like Kinase 1 (DCLK1), Musashi RNA Binding Protein 1 (MSI1), and Leucine Rich Repeat Containing G Protein-Coupled Receptor 5 (LGR5), which have been reported to take part in both normal intestinal homeostasis and GI-tumorigenesis, as summarized in Table S1.

This study investigates the SC phenotypes of spontaneous, low-LET, and high-LET (^56^Fe-ion) radiation-induced tumors (adenomas) in *Apc*^1638N/+^ mice. We also compared the SC phenotypes in the adjacent normal tissue. Here, we demonstrate that HZE-induced tumors display higher SC heterogeneity and enhanced oncogenic β-catenin expression, which has implications for understanding the higher risk of carcinoma after space radiation exposure.

## 2. Materials and Methods

### 2.1. Animal Experiment and Collection of the Samples

Six- to eight-week-old male *Apc*^1638N/+^ mice were maintained, irradiated, and euthanized as previously described [5,7,9]. All procedures were performed according to the approved animal protocols from the Institutional Animal Care and Use Committee at Georgetown University (Protocol#2016-1129; date 12 October 2021) and at Brookhaven National Laboratory (Protocol#345, date 1 August 2021). Briefly, the mice were bred and genotyped at GU and shipped to BNL for irradiation at the NASA Space Radiation Laboratory (NSRL). Mice were subjected to whole-body irradiation at a dose of 0.5 Gy ^56^Fe (energy: 1000 MeV/n; LET: 148 keV/μm) or γ-rays (^137^Cs source), and an age-matched control group received no radiation. The mice were sacrificed by CO_2_ asphyxiation 150 days after being irradiated. The small intestine was surgically removed, cleaned with buffered saline, fixed with formalin, processed for sectioning (5 μm thickness), and hematoxylin/eosin (H&E) staining. H&E-stained tissue slides were scored for premalignant (adenoma) and malignant tumor (carcinoma) by a board-certified pathologist.

### 2.2. Immunohistochemistry

**Figure 1:**
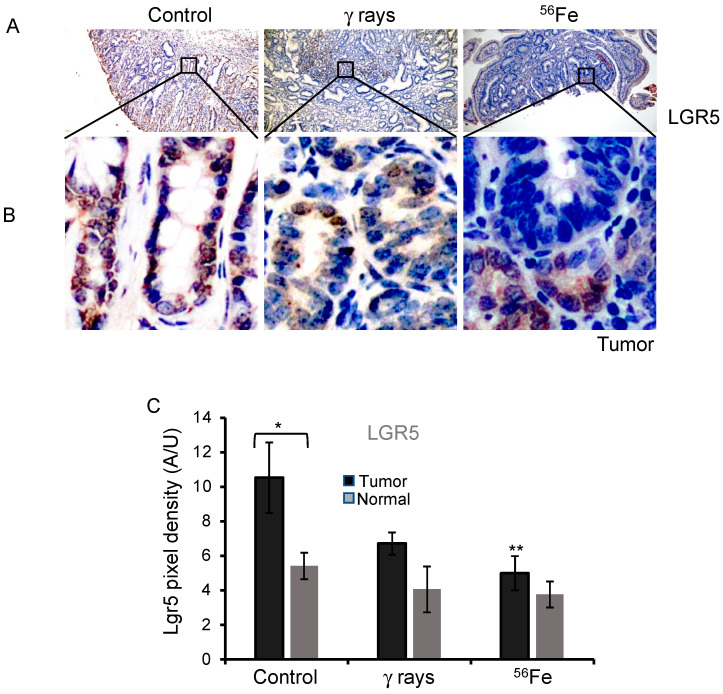
Detection of LGR5 expression in intestinal tumors after irradiation. Sample images of spontaneous, γ-rays, and ^56^Fe-irradiated intestinal tumor cells (**A**,**B**) stained for LGR5. (**C**) The bar graph represents the average DAB pixel density of LGR5 in all images taken of spontaneous, γ-rays, and ^56^Fe-irradiated tumor and normal tissue. The nuclei were counterstained with hematoxylin in blue. Scale bar tumor: 100 μm. *, significant relative to normal tissue, **, significant relative to spontaneous tumor sample. Statistical significance is set at *p* < 0.05 error bars represent mean ± SEM.

**Figure 2:**
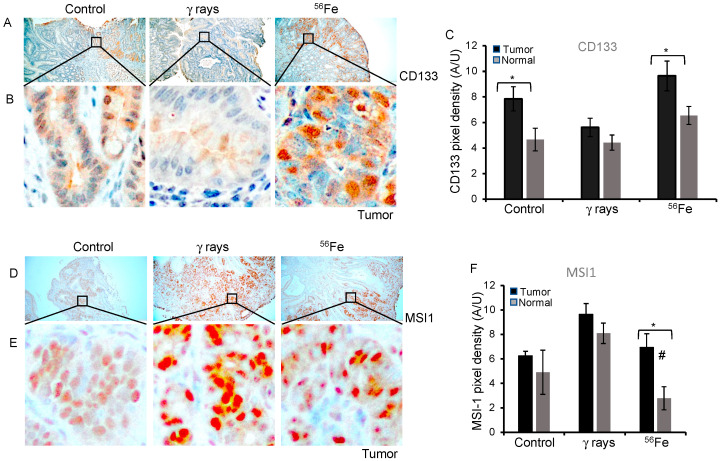
Differential response of CD133 and MSI1 after low- and high-LET irradiation in intestinal tumor. Sample images (**A**,**B**) of spontaneous, γ-rays, and ^56^Fe irradiated intestinal tumor cells stained for CD133 in dark brown DAB. **(C**) DAB pixel density was quantified from at least 20 different fields of view, statistically analyzed, and graphically represented. Bar graph represents the average DAB pixel density of CD133 in all images taken of control, γ-rays, and ^56^Fe-irradiated tumor and normal tissue. Sample images (**D**,**E**) of spontaneous, γ-rays, and ^56^Fe irradiated tumor intestinal cells stained for MSI1 in dark brown DAB. (**F**) DAB pixel density was quantified from at least 10 different fields of view, statistically analyzed, and graphically represented. Bar graph represents the average DAB pixel density of MSI1 in all images taken of unirradiated control, γ-rays, and ^56^Fe-irradiated tumor and normal tissue. The nuclei were counterstained with hematoxylin in blue. Scale bar tumor: 100 μm. *, significant relative to normal tissue, #, significant relative to γ-ray tumor sample. Statistical significance is set at *p* < 0.05 error bars represent mean ± SEM.

**Figure 3:**
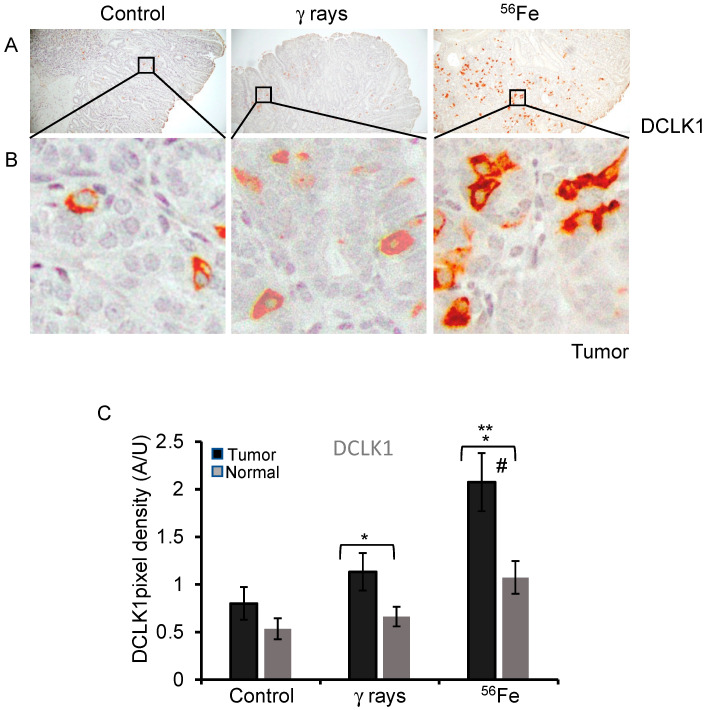
Response of DCLK1 after low- and high-LET irradiation in intestinal tumor. (**A**,**B**) Sample images of spontaneous, γ-rays, and ^56^Fe-irradiated tumor stained with DCLK1 in dark brown DAB. DAB-positive cells were counted from at least 20 different fields of view, statistically analyzed, and graphically represented. (**C**) Bar graph represents an average number of DCLK1-positive cells per 10× field. Higher DCLK1 positive cells were observed in radiation-induced tumors compared to the control. The nuclei are stained with hematoxylin in blue. Scale bar 100 μm. *, significant relative to normal tissue, **, significant relative to spontaneous tumor sample, #, significant relative to γ-ray tumor sample. Statistical significance is set at *p* < 0.05; error bars represent mean ± SEM.

**Figure 4:**
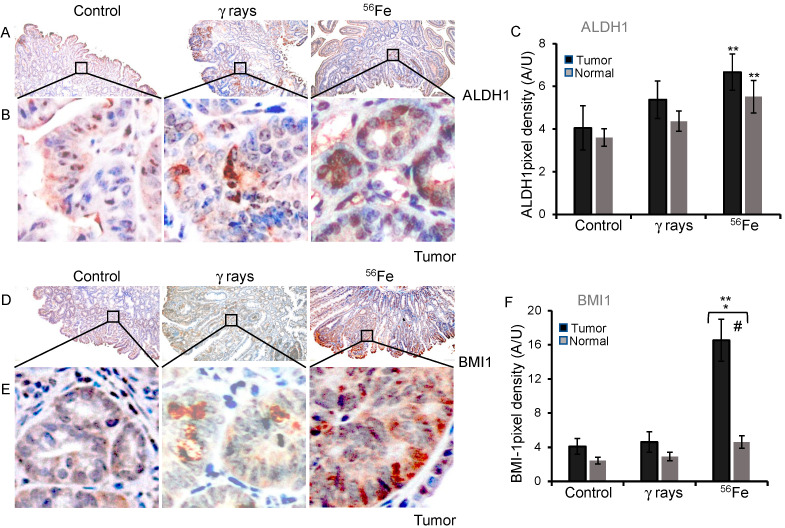
Enhanced ALDH1 and BMI1 expression in intestinal tumor cells after ^56^Fe-ions. (**A**,**B**) Representative images of spontaneous, γ-rays, and ^56^Fe irradiated intestinal tumor cells stained for ALDH1 in dark brown DAB. (**C**) Bar graph represents the average DAB pixel expression of ALDH1 in images taken of control, γ-rays, and ^56^Fe-irradiated tumor and normal tissue. (**D**,**E**) Sample images of spontaneous, γ-rays, and ^56^Fe-irradiated tumor stained with BMI1 in dark brown DAB after brightfield immunohistochemistry. (**F**) Average DAB expression of BMI1 staining in all images taken of spontaneous, γ-rays, and ^56^Fe-irradiated tumor and normal tissue. The nuclei are counterstained with hematoxylin in blue. Scale bar 100 μm. DAB pixel density was quantified from at least 25 different fields of view and statistically analyzed *, significant relative to normal tissue, **, significant relative to control sample, #, significant relative to γ-ray tumor sample. Statistical significance is set at *p* < 0.05; error bars represent mean ± SEM.

At least 15 premalignant tumors per group were used to perform immunostaining experiments as described earlier [5] in Figure 1, Figure 2, Figure 3 and Figure 4. The slides were prepared for immunostaining using deparaffinization, rehydration, and antigen retrieval. Finally, immunostainings were performed using a SuperPictureTM 3rd Gen IHC detection kit (Cat#87-9673; Invitrogen, Camarillo, CA) as per the manufacturer’s instruction. Briefly, the samples were blocked for endogenous peroxidase activity followed by incubation in protein-blocking buffer for 20 min. Tissue samples were then washed and incubated overnight at 4 °C with primary antibodies including ALDH1 (Cat#ab23375, Abcam, Cambridge, MA, dilution 1:100), BMI1 (Cat#NBP18732, Novus Biologicals, Littleton, CO, dilution 1:100), CD133 (Cat#PAB12663, Abnova, Taipei, Taiwan, dilution 1:100), DCLK1 (Cat#ab37994, Abcam, dilution 1:25), LGR5 (Cat#CF503316, Origene, Rockville, MD, dilution 1:100), MSI1 (cat#ab52865, Abcam, dilution 1:100), and β-catenin (Cat#9587S, Cell Signaling Technology, Danvers, MA, dilution 1:200). After multiple washes, samples were incubated with HRP-conjugated secondary antibody for 30 min, and signals were detected using 3,3’Diaminobenzidine (DAB) chromogen. Finally, sections were counterstained with hematoxylin, followed by permanent mounting and microscopic imaging. 

### 2.3. Fluorescence Microscopy

**Figure 5:**
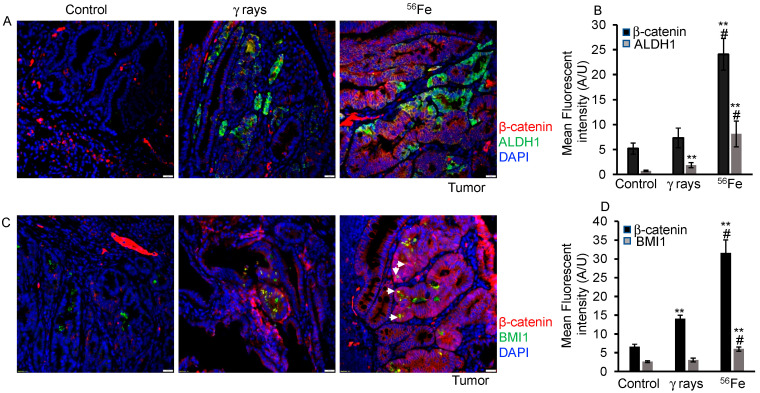
Enhanced β-catenin expression and nuclear localization coincide with higher BMI1 expression in ^56^Fe-exposed mice. (**A**) Sample images of spontaneous, γ-rays, and ^56^Fe-irradiated intestinal tumor after ALDH1 and β-catenin fluorescent immunostaining. The images show β-catenin (red), ALDH1 (green); nuclei with DAPI (blue). (**B**) Fluorescent intensity of β-catenin and ALDH1 in tumor cells was quantified from at least ten different FOV, and mean fluorescent intensity was calculated and graphically represented. (**C**) Representative images of BMI1 and β-catenin fluorescent immunostaining in tumors across the treatment group. Arrow marks show nuclear β-catenin expression in the area of BMI1 expression. (**D**) Bar graph showing the mean fluorescent intensity of BMI1 or β-catenin in intestinal tumors. Scale bar: 20 μm. **, significant relative to spontaneous tumor; #, significant relative to γ-ray tumor sample. Statistical significance is set at *p* < 0.05; error bars represent mean ± SEM.

The immunofluorescence experiment, Figure 5, was conducted using a minimum of 15 premalignant tumors from each treatment group, following the previously described protocol [5]. Briefly, deparaffinized slides were covered with the primary antibody solution. The primary antibodies used included β-catenin (Cat# SC-7963, Santa Cruz Biotechnology, Dallas, TX), BMI1 (Cat#NBP187321, Novus Biologicals), and ALDH1 (Cat# ab23375 Abcam), each diluted at 1:100. The slides were incubated with the primary antibodies overnight at 4 °C. The slides were taken out and washed three times in PBST. Appropriate fluorescence-labeled secondary antibodies were then applied for 1 h at room temperature (Alexa Flour 488 donkey anti-rabbit IgG (H + L) Cat#1796375, and Alexa Flour 594 donkey anti-mouse IgG (H + L) Cat#2069656, Invitrogen Life Technologies, Carlsbad, CA, USA). The slides were then mounted using a mounting medium containing DAPI for imaging.

### 2.4. Imaging, Quantification, and Statistical Analysis

To determine the specificity of the immunostaining, appropriate controls were run in parallel with the experimental samples. In each section, randomly chosen fields of vision for tumor lesions and normal mucosa were acquired by an observer blinded to the treatment groups using cellSens Entry v1.15 (Olympus Corp, Center Valley, PA, USA) for immunohistochemistry and immunofluorescence. Representative images including their scales were equally and proportionately reduced in size for figure compaction. For each treatment group, images were taken of the entire benign adenoma lesion and adjacent normal mucosa at 10× magnification. The respective SC marker expression in immunohistochemically stained digitalized images was analyzed by a semi-quantitative (DAB pixel density) method using ImageJ2 software fiji version: 2.14.0/1.54f [15,16]. Average DAB pixel density was measured from 20–25 images for each SC marker in tumors and normal intestine tissue sections. The fold change in DAB pixel density was calculated by dividing the pixel density of each treatment group by that of the spontaneous tumor pixel density. Immunofluorescence images, fluorescence intensity in the Cy3 channel, and FITC channel was measured by ImageJ2 fiji software. The statistical significance between the two groups was determined using a two-tailed paired Student’s *t*-test and a graphical presentation of the data show mean ± standard error of the mean (SEM). All data were statistically analyzed using and a *p*-value of 0.05 or less was applied for the detection of statistically significant differences.

## 3. Results

### 3.1. LGR5 Expression in ^56^Fe-Irradiated Mouse Intestinal Tumors and Normal Mucosa

To assess the expression of LGR5, IHC was performed in intestinal tissue sections containing premalignant tumor lesions from each group. This study focused on benign adenomas, which were confirmed by pathologic analysis. LGR5 expression was mostly localized at the crypt base in normal mucosa. As expected, cytoplasmic and membrane LGR5 stains were observed in the tumor and normal mucosa (Figure 1A,B and Figure S1A). The average LGR5 DAB staining in the normal mucosa for the control was 5.4 ± 0.76 (mean ± SEM), γ-rays 4.1 ± 1.33 (mean ± SEM), and ^56^Fe 3.77 ± 0.75 (mean ± SEM), indicating a decreasing trend in LGR5 expression after radiation exposure (Figure S1A). The decrease was not significant for γ-rays (*p* = 0.083) and ^56^Fe (*p* = 0.13) compared to the control. The average DAB pixel density for the spontaneous tumor tissue was 10.5 ± 2.04 (mean ± SEM), γ-rays 6.7 ± 0.64 (mean ± SEM), and ^56^Fe 5.0 ± 0.98 (mean ± SEM), also indicating a decreasing trend in LGR5 expression after radiation exposure. The decrease in level was insignificant for γ-rays (*p* = 0.82); however, it was significant in ^56^Fe (*p* = 0.026) compared to spontaneous tumors (Figure 1C). These results also demonstrate higher LGR5 expression in tumors than adjacent normal mucosa across the treatment group. Interestingly, radiation impedes LGR5 expression, and the effect was more pronounced after ^56^Fe than γ-rays (Figure 1A–C).

### 3.2. CD133 and MSI1 Expression in Tumors

CD133 expression was evaluated in the premalignant tumor lesions as well as adjacent normal mucosa in each treatment group. The longitudinal distribution mostly shows its expression in crypts, with diffuse cytoplasmic staining patterns observed in normal mucosa across the groups (Figure 2A,B and Figure S1B). Interestingly, distinct nuclear CD133 was detected in ^56^Fe tumors (Figure 2B). The average CD133 DAB pixel density in normal mucosa of the control was 4.67 ± 0.88 (mean ± SEM), γ-rays 4.4 ± 0.59 (mean ± SEM), and ^56^Fe 6.54 ± 0.71 (mean ± SEM) was observed, suggesting insignificant change after radiation ^56^Fe (*p* = 0.1) or γ-rays (*p* = 0.8). However, change was significant in ^56^Fe compared with γ-rays (*p* = 0.027). Analyzing the average DAB pixel density in spontaneous tumors was 7.85 ± 0.94 (mean ± SEM), γ-rays 5.61 ± 0.6 (mean ± SE), and ^56^Fe 9.64 ± 0.70 (mean ± SE), implying insignificant change after γ-rays (*p* = 0.065) or ^56^Fe (*p* = 0.24). These results indicate a significantly higher level of CD133 in the spontaneous tumors (*p* = 0.02) and ^56^Fe (*p* = 0.04) compared to adjacent normal mucosa (Figure 2C). MSI1 immunostaining showed cytoplasmic and nuclear expression in normal mucosa and tumors (Figure 2D,E and Figure S2A). The average MSI1 DAB staining in the normal mucosa for the control was 4.91 ± 1.8 (mean ± SEM), γ-rays 8.09 ± 0.84 (mean ± SEM), and ^56^Fe 2.78 ± 0.94 (mean ± SEM) (Figure 2F), indicating differential response with LET. The average DAB pixel density for the spontaneous tumor tissue was 6.25 ± 0.37 (mean ± SEM), γ-rays 9.63 ± 0.88 (mean ± SEM), and ^56^Fe 6.95 ± 0.94 (mean ± SEM). The results indicate a higher level of MSI1 in the tumors than in the normal mucosa of all treatment groups; however, the difference is particularly significant in ^56^Fe (*p* = 0.012). The data showed an increase in MSI1 in γ-rays compared to the control, while a decrease in response to ^56^Fe was observed (Figure 2F).

### 3.3. Detection of DCLK1 Expression in Irradiated Intestinal Tumors

The staining of DCLK1 expression mostly showed membrane and cytoplasmic staining in tumors and normal mucosa (Figure 3A,B and Figure S2B). DCLK1-positive cells were distributed across the crypt-villi axis. Average DAB pixel density in the normal mucosa of the control was 0.53 ± 0.11 (mean ± SEM), γ-rays 0.66 ± 0.1 (mean ± SEM), ^56^Fe 1.07 ± 0.172 (mean ± SEM), indicating an increase in DCLK1 positivity with high LET. The increase was significant for ^56^Fe (*p* = 0.006) compared to the control normal mucosa. DCLK1 expression in the spontaneous tumors was 0.8 ± 0.172 (mean ± SEM), γ-rays 1.13 ± 0.19 (mean ± SEM), and ^56^Fe 2.08 ± 0.3 (mean ± SEM), depicting an increasing order of expression with ^56^Fe. The increased tumor DCLK1 expression was significant in ^56^Fe relative to γ-rays (*p* = 0.012) or to spontaneous (*p* < 0.001) tumors (Figure 3C). Moreover, the data indicate higher levels of DCLK1 expression in tumors relative to adjacent normal mucosa across the groups, and it was significant for γ-rays (*p* = 0.039) and ^56^Fe (*p* = 0.006).

### 3.4. Assessment of ALDH1 and BMI1 Expression in Low- and High-LET Induced Tumors

As expected, ALDH1-positive cells are mostly localized at the crypt base in normal mucosa (Figure S3A). The staining pattern shows cytoplasmic as well as nuclear expression in the tumors (Figure 4A,B). The quantification of the ALDH1 DAB staining in normal mucosa for the control was 3.6 ± 0.41 (mean ± SEM), γ-rays 4.4 ± 0.47 (mean ± SEM), and ^56^Fe 5.5 ± 0.75 (mean ± SEM), whereas in the spontaneous tumors, it was 4.05 ± 1.03 (mean ± SEM), γ-rays 5.4 ± 0.88 (mean ± SEM), and ^56^Fe 6.7 ± 0.85 (mean ± SEM), indicating higher expression after radiation exposure (Table 1). The result was significant for ^56^Fe (*p* = 0.03, normal mucosa; *p* = 0.046, tumors) compared to the control (Figure 4C). BMI1 was mostly cytoplasmic and localized at the crypt base in normal mucosa (Figure S3B), whereas in tumors it showed cytoplasmic and nuclear expression (Figure 4D,E). The quantification of the BMI1 DAB staining in tumors and normal mucosa is summarized in Table 1, indicating higher expression after radiation exposure.

DAB pixel density indicates a substantial increase in BMI1 expression in the ^56^Fe-irradiated tumor (four-fold, *p* < 0.01) and normal intestine (two-fold, *p* = 0.012) relative to the spontaneous or unirradiated normal mucosa, respectively. This relative increase is the most pronounced of all of the SC markers tested (Figure 4F).

### 3.5. Evaluation of β-Catenin Expression and Nuclear Localization in HZE-Induced Tumors

To further identify subpopulations of cells with higher stemness features, we performed immunofluorescent co-staining experiments between two markers (ALDH1 or BMI1) and nuclear β-catenin in tumors. We found that in normal mucosa, positive cells were present as single or multiple cells in the crypts, while in tumors, there was an overexpression of the markers (Figure 5). Analysis of β-catenin expression in intestinal tumors showed a 1.5-fold increase in γ-rays and a 4-fold increase in ^56^Fe compared to spontaneous tumor tissue (Figure 5B,D). Additionally, ALDH1-positive cells showed increased β-catenin expression in irradiated samples relative to the control. Moreover, the tumor area containing ALDH1-positive cells demonstrated higher β-catenin expression, but with limited or no nuclear localization in irradiated samples (Figure 5A). Interestingly, fields with BMI1-positive cells showed higher β-catenin expression as well as nuclear localization after ^56^Fe irradiation compared to γ-rays or spontaneous tumors (Figure 5C).

## 4. Discussion

SC accumulation is known during tumorigenesis in mice as well as in humans, and the progressive buildup of stem-like cells is known to drive carcinoma development [17]. Additionally, crypt SC overpopulation in *Apc*-mutant mice has also been reported during intestinal tumor development [18]. Using the SC markers listed in Table 2, we demonstrated the differential accumulation of SC after low- and high-LET radiation exposure in *Apc*^1638N/+^ mice intestinal tumors. Notably, high-LET IR exposure resulted in a significant increase in SC markers (CD133, DCLK1, ALDH1, and BMI1) in tumors as well as in the normal intestinal mucosa, relative to low-LET exposure. Contrary to high-LET, MSI1 expression was highest after low-LET IR exposure. A LET-dependent decrease in LGR5 expression and increase in DCLK1 expression was also noted (Table 2). Moreover, increased expression of BMI1 and ALDH1 coincided with the activation of oncogenic β-catenin signaling.

LGR5-positive SC has been implicated in both normal GI homeostasis and tumor development [19]. Notably, LGR5 functions as a tumor suppressor during colon cancer progression [20,21,22]. IR exposure is known to reduce the number of LGR5-positive cells in the intestine owing to the higher radiosensitivity of these cells [23]. Additionally, LGR5 expressing cells readily undergo senescence after sub-lethal high-LET radiation exposure [8]. In concurrence, a LET-dependent decrease in LGR5 positive SC in both normal and tumors was observed, which suggests the loss of tumor suppressive effects of LGR5 in ^56^Fe-induced tumors. Additionally, reduced levels of LGR5 have been found to be associated with aberrant Wnt signaling and β-catenin expression [24]. Crypts deficient in LGR5-positive cells are competent to undergo hyperplasia upon loss of APC. These data argue that Lgr5-negative reserve stem cells are radiosensitive (or at least decrease as tumors develop) and that LGR5-positive cells are crucial for robust intestinal regeneration following radiation exposure, but are dispensable for premalignant hyperproliferation [25].

CD133 levels in colorectal cancers have been found to increase in a radiation dose-dependent manner [26]; an elevated CD133 level has been implicated in the development of resistance to chemoradiotherapy [27], and has been linked to poor prognosis in GI cancers [28]. Notably, our study showed increased levels of CD133 in high-LET irradiated normal mucosa as well as in tumor tissue compared to unirradiated samples. Whereas, in tumor tissue, CD133 was significantly higher in both the spontaneous and high-LET compared to the low-LET group. These results suggest differential effects of low-and high-LET radiation on CD133 expression in intestinal tumors. Moreover, loss of APC in mouse IEC is known to facilitate higher expression of CD133 in mouse intestine [29]. MSI1 is a known critical modulator that promotes the development of colorectal cancer stem cells (CSC) and also enhances CRC chemoresistance [30]. Additionally, studies have shown that ionizing radiation can increase the expression of MSI1 in GI-stem cells [31], suggesting that space radiation may increase the risk of GI cancer. DCLK1-expressing cells in the intestinal epithelium play a crucial role in DNA damage response and cell survival after radiation-induced injury [32,33], and are implicated in decreasing radiosensitivity and sustaining tumor progression [34]. Moreover, a study showed long-lived DCLK1-positive cells maintain quiescence even following oncogenic mutation, but were activated by tissue injury to initiate colon cancer [35]

BMI1 overexpression led to increased repair and resistance to radiation [36]. In fact, our data suggested that high-LET radiation is more effective at inducing ALDH1 and BMI1 expression, while lower LET radiation (γ-rays) is less effective. Furthermore, evidence suggests that the expression of ALDH1 or BMI1 is affected by the local microenvironment, age, tumor size, and type. This finding is significant in radiation-induced carcinogenesis, as higher ALDH1 expression may be associated with increased cancer risk.

We found an increase in nuclear β-catenin in ^56^Fe-induced intestinal tumors compared to normal tissue or γ-rays irradiated intestinal tumors. Studies have previously demonstrated that β-catenin expression and nuclear localization rise after high-LET radiation in intestinal tumors [9]. One study suggested that *Apc* mutation derails ALDH-positive cell maturation and facilitates cancer recurrence and development after radiation therapy [37]. Research has shown an association between BMI1 and β-catenin in colon cancer, explicitly showing increased BMI expression through the Wnt/β-catenin signaling pathway [38]. This relationship between BMI1 and β-catenin helps to support our findings that BMI1 and nuclear β-catenin are colocalized and more highly expressed in high-LET-induced intestinal tumors [11]. The correlation of increased nuclear β-catenin and increased tumor SC markers may suggest a potential pathway by which these markers are related to the increased tumor growth. Additionally, β-catenin has been shown to be upregulated in several types of tumors, and its increased expression has been associated with increased tumor aggressiveness [39,40]. The upregulation of stemness features in premalignant tumors exposed to particle radiation suggests that HZE-ion radiation has an increased likelihood of carcinoma development, and the expansion and persistence of stem-like tumor cells may influence their frequency. However, the functional implications of these markers and the mechanism involved in controlling space radiation-induced cancer development require further investigation.

## 5. Conclusions

The present study demonstrated altered expression profiles of several ISC markers (ALDH1, BMI1, CD133, DCLK1, LGR5, and MSI1) and β-catenin in space radiation-induced intestinal premalignant tumors and adjacent normal mucosa in the *Apc*^1638N/+^ mice model. These findings may have implications for estimating cancer risk after space radiation exposure.

## Figures and Tables

**Table 1:** Average DAB pixel density of ALDH1 and BMI1 in tumor and normal intestine tissues.

ISC Marker	Tissue	Sham	γ-rays	^56^Fe
ALDH1	Tumor	4.05 ± 1.04	5.37 ± 0.88	6.66 ± 0.85
	Normal	3.61 ± 0.41	4.37 ± 0.48	5.51 ± 0.76
BMI1	Tumor	4.1 ± 0.93	4.62 ± 1.19	16.53 ± 2.46
	Normal	2.43 ± 0.41	2.91 ± 0.51	4.60 ± 0.73

**Table 2:** Fold change in SC expression relative to the spontaneous tumor in low- and high-LET induced intestinal premalignant tumors.

ISC Marker	Spontaneous	γ-rays	^56^Fe
ALDH1	1	1.33	1.65 *
CD133	1	0.71	1.23
MSI1	1	1.5	1.1
BMI1	1	1.1	4.0 *
DCLK1	1	1.4	2.6 *
LGR5	1	0.64	0.48 *

NOTE: * Significant change in HZE-induced GI-tumor relative to spontaneous tumors by immunohistochemistry (Figure 1, Figure 2, Figure 3 and Figure 4).

## Data Availability

All data is contained within the article.

## Supplementary Materials

The following supporting information can be downloaded at: https://www.mdpi.com/article/10.3390/cancers15174240/s1, Figure S1: Immuno-detection of Lgr5 or CD133 in the normal mucosa of *Apc*^1638N/+^ intestine; Figure S2: Immuno-detection of MSI1 and DCLK1 expression in intestinal normal mucosa of *Apc*^1638N/+^ mice; Figure S3: Expression analysis of ALDH1 and BMI1 in normal intestinal crypts in *Apc*^1638N/+^ mice after irradiation; Table S1: Description of SC markers and some of their roles in normal tissue homeostasis and GI carcinogenesis. References [11,17,37,41,42,43,44,45,46,47,48,49,50] are cited in the supplementary materials.
