# Supplementary material for: Expression of Stem Cell Markers in High-LET Space Radiation-Induced Intestinal Tumors in Apc1638N/+ Mouse Intestine
Source: Cancers (Basel). 2023 Aug 24;15(17):4240. doi: 10.3390/cancers15174240 (PMC10486545; doi:10.3390/cancers15174240)
Supplement: Supplementary file 1 [file cancers-15-04240-s001.zip › cancers-2543176-supplementary.pdf]

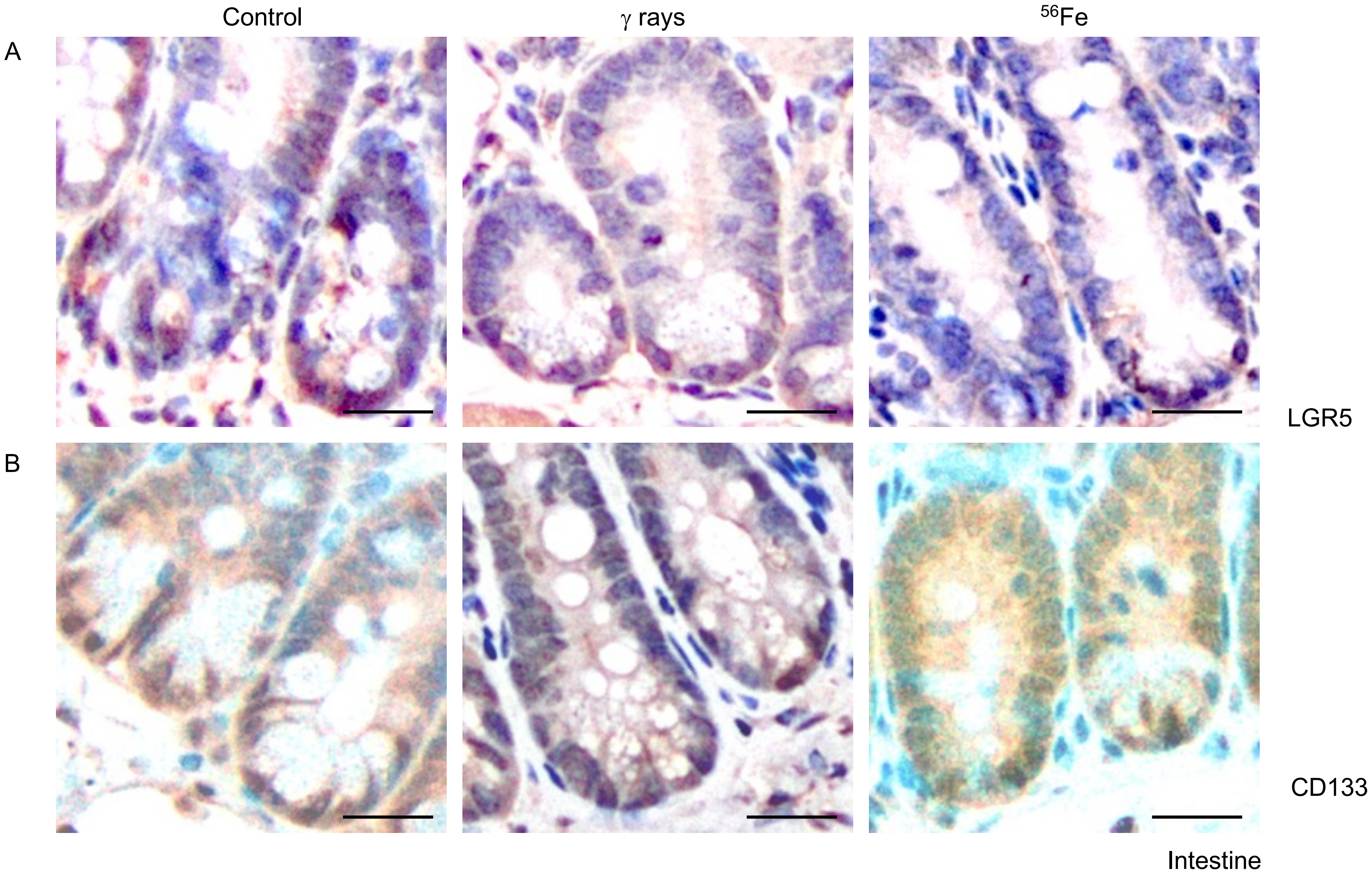

Figure S1: **Immuno-detection of Lgr5 or CD133 in the normal mucosa of *Apc*$^{1638N/+}$ intestine**. A) Representative image of LGR5 staining in intestinal normal mucosa. B) Sample images of CD133 staining in the normal intestinal mucosa. Nuclei were counterstained with hematoxylin in blue. Images were taken at 10X magnification. Scale bar 20μm

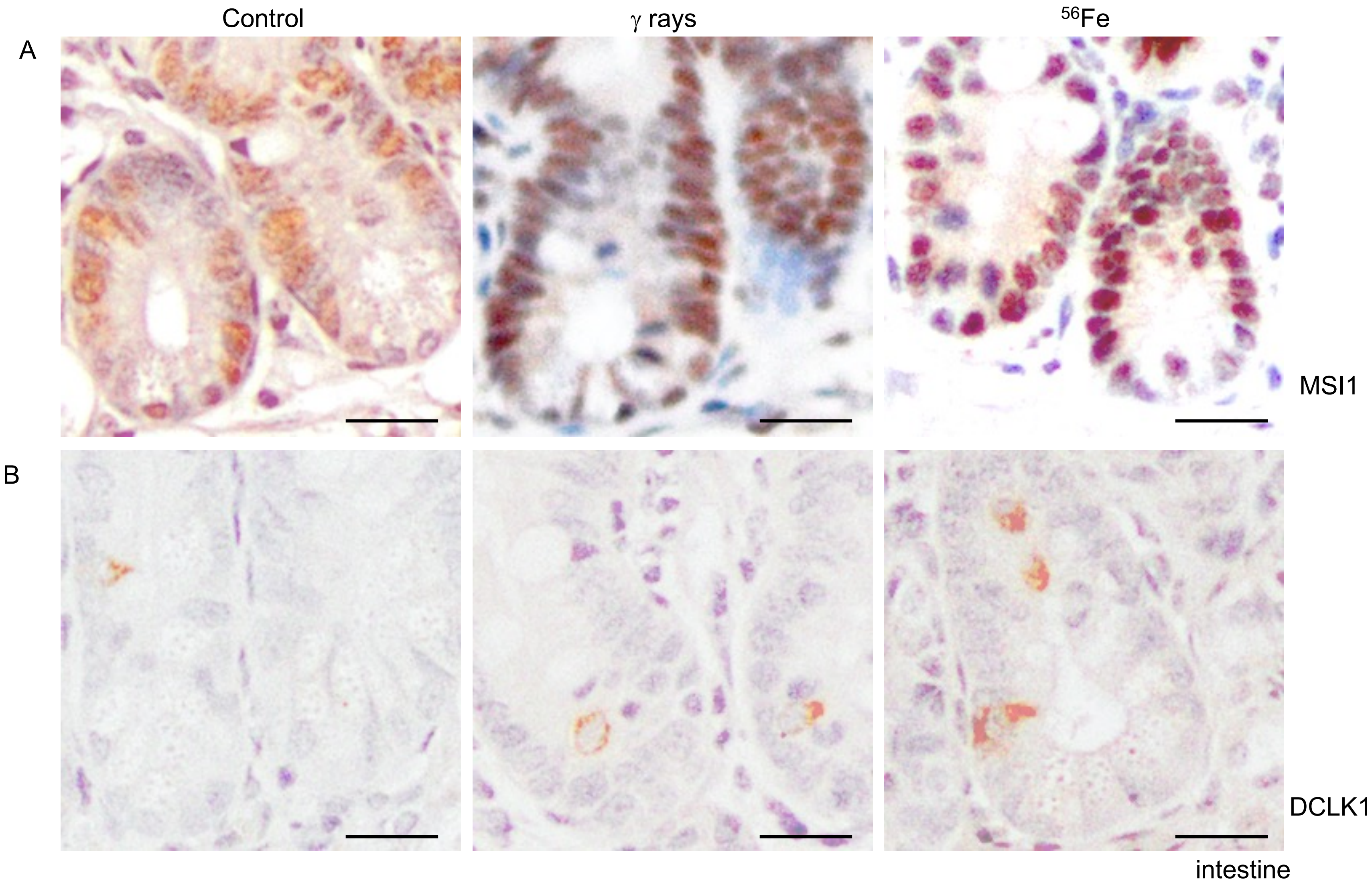

Figure S2: **Immuno-detection of MSI1 and DCLK1 expression in intestinal normal mucosa of *Apc*$^{1638N/+}$ mice.** A) Sample images showing MSI1 staining in the intestinal crypts after irradiation. B) Representative images showing DCLK1 expression in intestine after irradiation. Nuclei were counterstained with hematoxylin in blue. Images were taken at 10X magnification. Scale bar 20μm

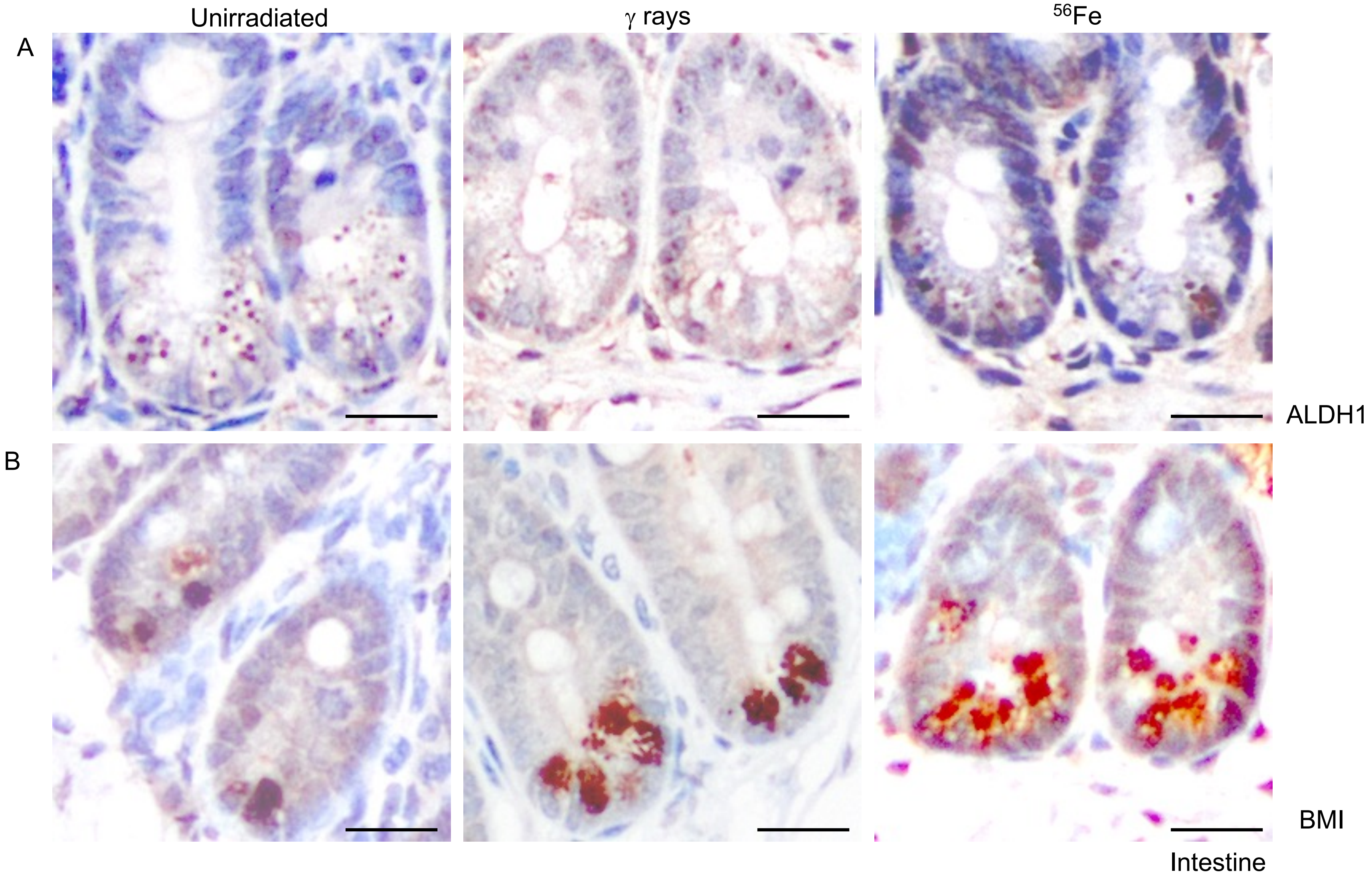

Figure S3 **Expression analysis of ALDH1 and BMI1 in normal intestinal crypts in *Apc*$^{1638N/+}$ mice after irradiation.** A) Illustrative image of ALDH1 expression in intestinal crypt after irradiation. B) Images showing BMI1 detection in normal intestinal crypt after irradiation. Nuclei were counterstained with hematoxylin in blue. Images were taken at 10X magnification. Scale bar 20μm

Table S1. Description of SC markers and some of their roles in normal tissue homeostasis and GI carcinogenesis.

SC marker	Roles in GI biology and tumor development
ALDH1	Reliable marker for normal and malignant human colonic stem cells [17]; APC loss results in an increase of ALDH expression; APC mutation causes the ALDH+ SC to remain immature (reduced differentiation) [37]
BMI1	Molecular marker of multipotent adult stem cells in the small intestine [41]; Promotes regeneration of the intestinal epithelium and represents the cells-of-origin in intestinal cancer [11]; Bmi-1 promotes invasion and migration of cancer SC through the downregulation of E-cadherin [42].
CD133	Marks stem cells in small intestine susceptible to transformation into tumors [43]; maintains homeostasis and intestinal regeneration; upregulated in colon tumorigenesis [44].
DCLK1	A stem cell marker, regulates pro-survival signaling and self-renewal of intestinal tumor cells [45]; promotes colorectal cancer stemness, aggressiveness [46], and epithelial-mesenchymal transition [47].
MSI1	Constitutive expression impairs mouse postnatal development and intestinal homeostasis [48]; plays major role in intestinal epithelium renewal and colon cancer development [49].
LGR5	Molecular marker of multipotent adult stem cells in the small intestine, promotes regeneration of the intestinal epithelium [50]; functions in the cells-of-origin in intestinal tumors [11].
